# Taxing highly processed foods: What could be the impacts on obesity and underweight in sub-Saharan Africa?

**DOI:** 10.1016/j.worlddev.2019.03.006

**Published:** 2019-07

**Authors:** Ole Boysen, Kirsten Boysen-Urban, Harvey Bradford, Jean Balié

**Affiliations:** a School of Agriculture & Food Science and Geary Institute for Public Policy, University College Dublin, Ireland; b Department of International Agricultural Trade & Food Security, University of Hohenheim, Stuttgart, Germany; c Foreign Agricultural Service, United States Department of Agriculture (USDA), Ottawa, Canada; d Agri-food Policy Platform, International Rice Research Institute, Los Baños, Laguna, Philippines

**Keywords:** Highly processed foods, Obesity, Underweight, Food taxes, Trade, Sub-Saharan Africa

## Abstract

The consumption of highly processed food has been singled out as one of the factors responsible for the rapidly increasing prevalence of obesity and its associated non-communicable diseases and costs. While obesity prevalence is still comparatively low in lower-income sub-Saharan Africa (SSA), development prospects in this region render markets especially attractive for these foods, whose consumption is already growing at higher rates than in developed countries. This might be reflected in the massive rise in obesity prevalence growth rates in SSA over the past decade, while many of these countries are simultaneously struggling with high undernutrition prevalence.

Using a newly constructed cross-country panel dataset, this study econometrically investigates the effect of higher import tariffs on highly processed *vis-à-vis* less-processed foods with respect to their impacts on obesity and underweight prevalence in the adult population. While the analysis is global, the discussion focuses primarily on SSA. The effects of the tariff differences are found to be significant and substantial and to differ by income level of the country as well as by gender. More generally, the results show that policies affecting the consumer price differential between the two food groups are effective in influencing obesity and underweight prevalence and that these two issues cannot be treated separately.

## Highlights

• Estimating effects of highly processed food (HPF) taxes on obesity and underweight.
• Trade liberalization mitigates underweight but aggravates obesity prevalence in SSA.
• Taxes on HPF reduce obesity, increase underweight but effects differ by region, income, gender.
• Magnitude of consumption taxes needs to be very substantial to have a notable effect.
• Integrated policy framework considering both issues simultaneously is required.

## 1 Introduction

The rapid growth in prevalence of obesity has long been recognized as a serious global problem and has even been called an epidemic (IFPRI, 2016, WHO, 2003, Townsend et al., 2016). Paradoxically, this problem is not exclusive to the developed world but also prevalent in developing countries, which are simultaneously challenged by persistent problems of undernutrition. In 2013, the share of adults considered overweight worldwide grew to 36.9 percent for men and 38.0 percent for women, with developing countries accounting for 60 percent of global obesity prevalence (Ng et al., 2014). Overweight (body mass index [BMI] of 25–30 kg/m^2^), and particularly obesity (BMI > 30 kg/m^2^), is associated with reduced overall health, loss of productivity and the development of an array of non-communicable diseases (NCDs) such as diabetes, cardiovascular diseases and cancer, and correspondingly with substantial health and economic costs^2^.

Obesity prevalence rises with economic development; obesity is mainly the result of a continued excess of energy intake over expenditure. As countries develop economically, not only are incomes rising – allowing the consumption of more calories while expending less physical energy – but also diets are shifting from staple foods towards, for example, more animal products, fats and sugar, as well as more highly processed and convenience foods (Kearney, 2010). This culminates in the developed-country or “Western” diet characterized by higher intake of free sugars, refined carbohydrates and animal-source foods and fats and is nutritionally imbalanced, as consumers tend to ingest excess calories and insufficient levels of micronutrients. This shift in diet is seen as one of the primary nutrition-related causes of obesity (see, e.g. Popkin & Gordon-Larsen, 2004) and dietary patterns in developing countries are indeed becoming increasingly similar to the Western diet (Popkin, Adair, & Ng, 2012) with an accompanying rise in obesity prevalence.

An important role in the emergence of nutrient imbalance and thus of the obesity epidemic is attributed to the shift in consumption towards highly processed foods^3^ (see, e.g. Hawkes, 2005, Moubarac et al., 2013, Popkin et al., 2012), which are often energy-dense but micronutrient-poor. These foods are nevertheless highly popular with consumers due to a multitude of (perceived) positive attributes, such as short or no preparation time, high palatability, ease of storage and transport, long shelf-life, lower price relative to less processed foods, improved food safety, or low cost per calorie. Consequently, while highly processed foods are part of the cause for the obesity problem, they might also be part of an answer to the undernutrition problem (Augustin et al., 2016).

Highly processed foods are very profitable (Monteiro et al., 2013, Stuckler et al., 2012) but their markets in developed countries are largely saturated (Reardon, Timmer, Barrett, & Berdegue, 2003). By contrast, countries in sub-Saharan Africa (SSA) continue to develop rapidly and promise strong growth in consumption and higher profits. Hence, multinational food companies are increasingly targeting SSA markets and intensifying their marketing efforts. Continued trade and investment liberalization create an environment conducive for foreign direct investment (FDI) and exploitation of global supply chain logistics which facilitate the expansion of supermarket chains, thereby increasing the availability and lowering the prices of highly processed foods (Reardon et al., 2003).

Indeed, Stuckler et al. (2012) observed that multinational food companies have already penetrated middle-income markets to a similar extent as they have in high-income countries. Using survey data from towns in Central Kenya, Demmler, Ecker, and Qaim (2018) found that individuals shopping in supermarkets are consuming larger shares of dietary energy from highly processed foods than individuals who do not. According to Moodie et al. (2013) and Stuckler et al. (2012), consumption of highly processed foods is, in fact, already growing at much higher rates in low- and middle-income than in high-income countries. Studying four SSA countries, Holmes et al. (2018) found evidence that a diet containing a high consumption level of processed food is associated with much higher risk of being overweight or obese.

Furthermore, undernutrition is still a widespread problem in SSA and it is known that *in utero* and childhood undernutrition increases susceptibility to obesity in later life (Nettle et al., 2017, Popkin et al., 2012). This constellation of factors in SSA could imply that with progressing economic development the obesity problem might become even more severe for current and future generations in SSA than what has been observed in developed countries until now.

Consequently, policies to discourage the consumption of highly processed foods to counteract the rise of obesity might have more value the sooner they are implemented. Moreover, they need to be integrated with the policies to combat undernutrition.

In efforts to reduce obesity prevalence and associated NCDs, developed and developing countries alike have been experimenting with an array of measures to counter the epidemic through channels such as education and information (e.g. food labelling, nutrition education, dietary guidelines, school physical activity programmes), price incentives (food or food content taxes and subsidies) and regulation (marketing targeted at young people, food re-formulation, school food); see Alston, MacEwan, and Okrent (2016) or Hyseni et al. (2017) for recent overviews.

Many countries have adopted or are considering consumption taxes or subsidies on specific food groups or contents to discourage unhealthy eating or encourage healthy eating, as reviewed in Thow et al. (2018). A frequently tax-targeted food group is sugar-sweetened beverages (SSBs), such as soft drinks, juices or sweetened milks. This is a narrow, well-defined group, believed to substantially contribute to the obesity problem, and the effects of corresponding taxes have been the subject of many research studies; see Cornelsen and Smith (2018) for an overview. Other recent studies particularly focus on the effect of different policies on the development of overweight and obesity. Usually, these studies are country-specific and focus on more developed countries (see, e.g., Wright, Smith, & Hellowell, 2017). For example, Caro, Ng, Taillie, and Popkin (2017) provide a simulation exercise to analyse the effect of different policy options to reduce the consumption of unhealthy foods in Chile, which is stated to be the country with the highest consumption of SSBs and fast food. While Batis, Rivera, Popkin, and Taillie (2016) evaluated the effect of a tax on non-essential energy dense foods imposed in Mexico, Stacey, Tugendhaft, and Hofman (2017) assess the impact of SSB taxation in South Africa both utilizing consumption demand models. In these country-specific studies national survey data are used to investigate the effects of taxing specific food groups, such as SSBs or energy-dense foods (EDFs), or ingredients, such as sugar or fat, applying a broad range of different quantitative and qualitative methodologies. Extensive reviews of the available literature quantifying the effects that taxes and subsidies on various classifications of foods and nutrients have on consumption and on health behaviour and outcome are provided by, for instance, Hagenaars et al., 2017, Niebylski et al., 2015, Wright et al., 2017 or Backholer et al. (2016) with a focus on weight gains according to socio-economic position, or by Kirkpatrick, Raffoul, Maynard, Lee, and Stapleton (2018) with a focus on sugar consumption.

However, the consumption of the entire broad group of highly processed foods is suspected to promote obesity – see, for example, Hawkes, 2005, Monteiro et al., 2013, Moubarac et al. (2013) and Thow, Jan, Leeder, and Swinburn (2010) – but taxes on such broad groups have received little attention, likely due to the lack of suitable data. Moreover, the concurrent prevalence of both obesity and undernutrition – the double burden of malnutrition – in SSA creates a dilemma for policy-makers. Indeed, it is often the case that policies aiming at reducing undernutrition exacerbate the obesity problem and vice versa.

The present study builds on this literature and investigates the potential of taxes on highly processed foods or subsidies on less processed foods as a means for reducing obesity prevalence. At the same time, it examines what effect such policies might have on the other end of the bodyweight spectrum, on underweight prevalence. The main questions studied include: To what extent would it be possible to reduce obesity and underweight prevalence through taxes or subsidies that increase the price gap between unhealthy highly processed foods and healthy foods? What effects might further trade liberalization have on those prevalence rates? How big would such policy interventions need to be to have a notable impact? How would the effects of these taxes differ between genders?

In contrast to most other studies looking at the effects of taxing specific food groups on obesity using national survey data for individual countries, here we take a global perspective. We aim to contribute to the literature by analysing the impact on obesity and underweight of tax policies on the broad group of highly processed foods in a cross-country study with an assessment of the likely effects in SSA. As no consistent database for prices and taxes on the global level is available, we create a novel concordance between a classification system of foods and one of traded goods to exploit the data from an import tariff database which has been regularly and consistently updated and which now covers most countries of the world over a long time period.

Many countries employ import protection strategies, which cause differences in tariffs across products and according to level of processing of the products. One example is the so-called tariff escalation where the tariffs applied are the higher, the more processed the products are, see Regmi et al. (2005). Assuming pass-through of taxes applied at the national border to retail prices, such a tariff pattern results in policy-created price differences between unprocessed, basic foods and their processed counterparts, which discourages consumption of the latter and may thereby lower obesity prevalence. Studies analysing the effect of international trade and openness to trade on food security reveal, e.g., that reductions of tariffs on agricultural and food products reduce domestic market prices and hence increase food access of poor people (FAO, 2018, Matthews, 2014). Trade openness not only improves the availability and affordability of food but also contributes to increasing food choices for consumers. According to Traill (2017), changes in relative prices of foods caused by trade influence consumer choices. Thus, if, for example, tariff reductions decrease prices for processed foods versus other foods, this can change consumption patterns in a way that energy-dense, micro-nutrient poor products gain a greater share in overall calorie intake. Correspondingly, trade liberalization can affect diets through increased availability and lower prices of calorie-rich, nutrient-poor foods (Thow, 2009). Asfaw (2008) shows that diets of poor households in Guatemala are negatively affected by the expansion of supermarkets and the corresponding increase in availability of processed foods since particularly poor households tend to buy cheap and filling processed food items. As processed foods might serve as a beneficial source of cheap calories for the poorer population (Traill, 2017), policy-created price differences between highly processed foods and their less processed counterparts might increase underweight prevalence while obesity prevalence is reduced.

The results from a global econometric analysis are then used to assess the potential of tax policies on highly processed foods to affect obesity and underweight prevalence. On the one hand, such interventions are particularly problematic in SSA countries because of the simultaneous occurrence of over- and underweight prevalence. On the other hand, such policies are also potentially more effective there due to the higher responsiveness of low-income consumers for whom obesity prevalence is predicted to grow fastest (see Jones-Smith et al., 2012, Ziraba et al., 2009).

The remainder of the study is organized as follows. Section 2 introduces the data employed and the econometric approach. Section 3 then presents and discusses the econometric estimations and their results. Finally, Section 4 synthesizes the outcomes and draws policy conclusions.

## 2 Methodology and data

For this study we take an indirect approach to investigate the potential of tax policies to affect obesity related to processed food consumption. The reason for this approach is that availability of country-level data on consumption, consumer prices and consumption taxes is insufficient and frequently lacks the detail required to distinguish processed from unprocessed food items. Therefore, our strategy to empirically investigate this question is based on the fact that countries systematically differentiate their import tariff patterns between processed and unprocessed food products.

We assume that the effects of import tariffs on border prices transfer to the retail prices of both the imported products and their domestically produced counterparts. More precisely, we assume that the proportional change in a product’s price at the border created by an import tariff causes a change in its retail price at a local market of the same proportion: The “law of one price” predicts that, in absence of trade frictions and with price flexibility and free competition, identical products will be sold for the same price in different locations (Feenstra & Taylor, 2011). This is because any opportunity for arbitrage would be exhausted immediately by market participants, causing prices to converge. Allowing for trade and transport costs and applying the same arbitrage logic, prices must be identical after adjustment for these costs. For our analysis, we weaken this assumption and only require that the border price and the local market’s retail price remain in the same proportion after some price change at the border. Furthermore, we assume that all domestically produced foods have imported variants which are perfect substitutes so that consumers would always choose the cheaper of the two and thereby cause convergence of these prices. Under these assumptions, the tariffs create a price wedge between processed and unprocessed products on retail markets. Our analysis further assumes that consumers react to changes in the relative prices by substitution, here specifically to the relation between prices for processed and unprocessed foods. Consequently, a positive (negative) price wedge discourages (encourages) the consumption of processed *vis-à-vis* unprocessed foods and thereby affects obesity and underweight prevalence.

### 2.1 Food classification and data

The approach chosen has the advantage that data on import tariffs have been systematically collected by the World Trade Organization (WTO) and previously the General Agreement on Tariffs and Trade (GATT) since 1988 from all member countries. These data are available from the United Nations Conference on Trade and Development (UNCTAD) Trade Analysis Information System database (TRAINS, 2017) and cover the large majority of countries for the recent years. The data are classified according to the Harmonized Commodity Description and Coding System (HS)^4^ at the 6-digit level, which differentiates 1 300 agricultural and food products by various criteria, including the degree to which they have been processed.

Nevertheless, the HS classification has been designed for customs purposes so that here the HS food items need to be reclassified with respect to their obesity-related properties, i.e. according to their degree of processing and ingredient composition. The reason for the scarcity of data on processed foods consumption is partly found in the traditional focus of research on identifying the health effects of foods classified into groups based on either their botanical and animal species origin or their nutrient content, whereas no comprehensive classification of different food processing activities and their effects on foods and health is available yet (Fardet et al., 2015). However, five systems which classify foods according to processing have been used for health studies (Moubarac, Parra, Cannon, & Monteiro, 2014).

Of those five systems, here we adopt the rather recent NOVA classification system (Monteiro et al., 2016). It has previously been applied successfully, for example, in a study by Canella et al. (2014) to identify the association between consumption of ultra-processed foods and obesity in Brazil. The NOVA system classifies food into the following four groups: (NOVA 1) unprocessed or minimally processed foods, such as fresh or frozen fruits and vegetables; (NOVA 2) processed culinary ingredients, such as starches or syrups; (NOVA 3) processed foods which often have been processed to increase their durability and are usually recognizable as the original food, such as canned vegetables, tinned fish preserved in oil, salted nuts or cheese; and finally (NOVA 4) ultra-processed food and drink products. The latter might be described as foods engineered by recombining ingredients created through extraction from and refinement of food and other organic sources through physical, biological and chemical processes. They typically contain more than five ingredients. Examples are carbonated soft drinks, crisps and other sweet, fatty or salty snack foods. The differentiating quality of NOVA 4 relative to NOVA 3 processed food products is that the former incorporate substances that have previously been extracted. Some typical processing techniques applied to ultra-processed foods include extrusion, moulding and pre-processing. Also, any products in NOVA 1 to 3 categories but containing “cosmetic or sensory intensifying additives”, such as artificial sweeteners, are classified as NOVA 4.

It is worth noting that a classification according to the level of processing of food, such as the NOVA classification, is a crude proxy for grouping products with particular unhealthy properties (see Gibney, Forde, Mullally, & Gibney, 2017, for a critique on the NOVA classification.) Botelho, Araújo, and Pineli (2018) argue that it is not the number of steps or the intensity of processing but the list of ingredients which renders a food unhealthy. Fardet et al. (2015) add that certain processing operations can change the structure of food and thereby alter its nutritional and health potential.

The final reclassification maps 897 6-digit HS tariff lines, corresponding to all agricultural and food items suitable for human consumption, to the four NOVA categories. There are cases in which a HS tariff line includes products of multiple NOVA groups.^5^ In such cases the more detailed, HS-based, 10- and 8-digit nomenclatures of the United States (USA) and the European Union (EU) have been consulted to get a better indication of what is subsumed under these tariff lines.^6^ Ultimately, these lines were classified by personal judgement as to which of the potential NOVA groups might have a dominant value share in the particular HS line. This reclassification is applied to calculate a trade value-weighted import tariff average for each NOVA category and each country and year pair available in the TRAINS database. More specifically, the import values from the world into each country as well as the corresponding “weighted average” of the “effectively applied tariff” rate at the HS 6-digit level are utilized.

Since TRAINS reports import tariffs for the EU as a whole rather than of its member countries separately, we separate the EU import data into individual member countries using the UN COMTRADE (2017) database: for each member country and HS product code, we combine the EU’s common external tariff as reported in the TRAINS database with the individual country’s import value data from the COMTRADE database. In this way, we are able to recover valuable data for the diverse EU member countries.

Our main hypothesis is that the difference in prices between highly processed and less-processed products influences food consumption behaviour and thus related obesity and underweight prevalence. Hence, we calculate our main explanatory variable as the difference in the aggregate average trade-weighted tariff between the NOVA 4 and the NOVA 1 to 3 categories for each country and year.

The variable to be explained in our study is obesity prevalence measured as the percentage of the adult population aged 18 and above with a BMI equal to or greater than 30 kg/m^2^. The percentage of underweight prevalence, similarly defined by a BMI equal to or less than 18.5 kg/m^2^, is the second dependent variable adopted to examine the effects on the undernourished population.

This obesity and underweight data is taken from a study recently published in *The Lancet* by the NCD Risk Factor Collaboration (NCD-RisC, 2016). In a large-scale effort, NCD-RisC collected 1 698 population-based measurement studies and utilized those to estimate a complete and methodologically consistent large dataset covering 200 countries over the period from 1975 to 2014, using a Bayesian estimation approach. The BMI, obesity and underweight data provided by NCD-RisC were age-standardized to a World Health Organization (WHO)-defined standard population to allow age structure-independent comparability across countries. In the estimation, the authors also correct for the many differences between datasets, such as self-reported versus measured heights and weights, yielding a dataset comparable across countries. The female- and male-specific percentages taken from this dataset are combined into single values by using the share of females in the total population, as provided in the World Bank’s World Development Indicators (WDI) database (WDI, 2017). Note that, while BMI has emerged as a widely used standard to measure obesity and underweight because of the ease of obtaining the information for its calculation, it is not perfect as it does not differentiate, for example, between weight from fat or muscle, or among body shapes or genders.

A multitude of factors influence obesity prevalence. In order to derive a sound causal relationship between the NOVA-categorized import tariff measures and obesity prevalence, we control for factors which influence obesity and are potentially correlated with those tariff measures. The first of these is income per capita, proxied by GDP per capita in our study, as typically price elasticities of food demand change with the level of income, where higher incomes are accompanied with lower responsiveness to price changes. In addition, we add some of the main drivers of obesity to improve the precision of the estimates. These include the percentage of the population living in urban areas, the percentage of females engaged in wage labour, the percentage of the population aged 65 and above, and trade as a share of GDP. These statistics come from the WDI database (WDI, 2017). Indicators for information flows and cultural proximity have been retrieved from the KOF Index of Globalization^7^ (KOF, 2017), and the international Food Price Index (FPI) from the Food and Agriculture Organization of the United Nations (FAO) (FAO, 2017). The final variable is the percentage of the value of global NOVA 4 imports in total food imports. The choice of variables has been guided by theoretical considerations but also by data availability. Including variables such as education levels, healthcare, or percentage of employment in services would have greatly reduced the number of countries in the final dataset. While considered, controlling for healthcare quality proxied by health expenditure per capita has also been discarded due to potential endogeneity with the dependent variable and very high correlation with the GDP per capita variable.

The period from 2007 to 2013 has been chosen to construct a balanced panel covering as many countries as possible. In total, the final dataset represents 101 countries with complete data for all variables, as detailed in Table 1. The countries have been categorized by income group according to the World Bank’s classification^8^ and additionally cross-categorized by SSA or non-SSA country. The availability of the trade data is the bottleneck but has been improving over time. Variable descriptions and summary statistics are provided in Table 2. Additional summary statistics on the NOVA-categorized import tariff measures differentiated by income group and region are shown in Table A.1 in the Appendix.

**Table 1:** Countries included in the analysis, by income group.

Income group	Region	n	Countries
Low income (LI)	Non-SSA	2	Haiti, Nepal
	SSA	13	Benin, Burkina Faso, Burundi, Central African Republic, Gambia, Guinea-Bissau, Madagascar, Mali, Niger, Senegal, Togo, Uganda, United Republic of Tanzania

Lower middle income (LMI)	Non-SSA	17	Armenia, Bangladesh, Bolivia (Plurinational State of), Egypt, El Salvador, Georgia, Guatemala, Honduras, India, Indonesia, Jordan, Kyrgyzstan, Nicaragua, Pakistan, Philippines, Republic of Moldova, Ukraine
	SSA	5	Cabo Verde, Cameroon, Côte d’Ivoire, Kenya, Zambia

Upper middle income (UMI)	Non-SSA	19	Albania, Argentina, Belize, Bosnia and Herzegovina, Brazil, Bulgaria, Colombia, Costa Rica, Croatia, Cuba, Fiji, Guyana, Malaysia, Mexico, Panama, Paraguay, Russian Federation, The former Yugoslav Republic of Macedonia, Venezuela (Bolivarian Republic of)
	SSA	5	Botswana, Gabon, Mauritius, Namibia, South Africa

High income (HI)	Non-SSA	40	Australia, Austria, Bahrain, Belgium, Canada, Chile, Cyprus, Czech Republic, Denmark, Estonia, Finland, France, Germany, Greece, Hungary, Iceland, Ireland, Italy, Japan, Kuwait, Latvia, Lithuania, Luxembourg, Malta, Netherlands, New Zealand, Norway, Poland, Portugal, Qatar, Republic of Korea, Singapore, Slovakia, Slovenia, Spain, Sweden, Switzerland, United Kingdom, United States of America, Uruguay

Source: Own elaboration.

**Table 2:** Descriptive statistics.

		All			
Variable	Description	Mean	SD	Min	Max
% obese	Obese population (% of total)	16.6	8.2	1.9	36.7
% underweight	Underweight population (% of total)	5.5	5.9	0.7	27.7
${\text{dTariff}}_{N4-N123}$	NOVA 4 minus NOVA 1 to 3 import tariff	0.1	16.4	−110.0	187.3
${\text{dTariff}}_{N34-N12}$	NOVA 3 and 4 minus NOVA 1 and 2 import tariff	−0.5	13.9	−126.4	105.5
${\text{Tariff}}_{N123}$	NOVA 1 to 3 import tariff	12.2	13.7	0.0	146.0
${\text{Tariff}}_{N4}$	NOVA 4 import tariff	12.3	14.1	0.0	189.0
${\text{Tariff}}_{N12}$	NOVA 1 to 2 import tariff	12.4	14.6	0.0	157.1
${\text{Tariff}}_{N34}$	NOVA 3 to 4 import tariff	11.8	10.0	0.0	106.9
GDP/capita	GDP per capita (constant 2010 US$)/ 1000	17.5	21.7	0.2	112.0
% urban	Urban population (% of total)	62.3	21.8	9.9	100.0
% female labour participation	% of females labour participation	52.8	12.8	14.0	87.8
% trade/GDP	Trade (% of GDP)	91.7	57.1	22.1	441.6
% age ⩾65	Population ages 65 and above (% of total)	9.5	6.0	1.1	25.0
Information flows	Information flows index (KOF)	71.1	18.5	30.5	98.1
Cultural proximity	Cultural proximity index (KOF)	44.4	34.1	1.0	97.1
FPI	Global real food price index (FAO)	194.9	24.5	160.3	229.9
Global % ${\text{imports}}_{N4}$	Global NOVA 4 imports (% of total NOVA imports)	21.2	0.8	20.2	22.4

Source: Own computation.

### 2.2 Econometric specification

Our analysis employs a panel fixed effects estimation approach, as presented in the generally formulated Eq. (1).

(1) $$Y_{\mathrm{it}}=\tau T_{\mathrm{it}-1}+\overset{G}{\underset{g=1}{\sum }}\gamma_{g}(T_{\mathrm{it}-1}\times D_{\mathrm{ig}})+\overset{J}{\underset{j=1}{\sum }}\beta_{j}X_{\mathrm{jit}-1}+\overset{K}{\underset{k=1}{\sum }}\gamma_{k}Z_{\mathrm{kt}-1}+\alpha_{i}+{∊}_{\mathrm{it}}$$

In our base specification, $Y_{\mathrm{it}}$ represents obesity (or underweight) prevalence of country *i* in year *t*. $T_{\mathrm{it}}$ denotes our main variable of interest, the difference in tariffs between NOVA 4 and NOVA 1 to 3 (${\text{dTariff}}_{N4-N123}$). $T_{\mathrm{it}}$ is interacted with the dummy variable $D_{\mathrm{ig}}$ which is one if country *i* belongs to the country income and region group *g* (as specified in Table 1) and zero otherwise.

$X_{\mathrm{jit}}$ includes the set of *J* additional control variables which vary over countries and time. $Z_{\mathrm{kt}}$ is a set of *K* explanatory variables which control for global developments over time. $\alpha_{i}$ accounts for all country-specific fixed effects which are time-constant – in particular, geographic, institutional and cultural characteristics. Finally, ${∊}_{\mathrm{it}}$ is the error term.

More specifically, $X_{\mathrm{jit}}$ comprises the ln(GDP per capita) and ln(GDP per capita)^2^ to model the non-linear relationship between obesity and per capita income levels as one of the most important determinants of obesity. The percentage of urban population is included to account for better access to and availability of NOVA 4 foods in urban areas due to the density of shops and supermarkets and better transportation infrastructure. The share of the female population participating in the labour force is added to pick up effects arising from increased opportunity costs of working women and accounts for the reduced time required for meal preparation as a result of increased consumption of pre-prepared NOVA 4 foods. Furthermore, the share of the population age 65 and above indicates a population group which is associated with lower metabolic rates and lower levels of physical activity. Trade as a percentage of GDP is a proxy for trade openness, which increases availability and decreases prices of foods. Increased market access for multinational food companies could potentially result in lower prices for NOVA 4 relative to NOVA 1 to 3 products. The information flows index is composed of measures for Internet, television and newspaper use and thus represents a measure of marketing and international cultural exposure which might affect consumption preferences. The cultural proximity index is constructed from data on penetration of McDonald’s restaurants and IKEA stores as well as trade in books, and is supposed to measure the level of cultural convergence which might indicate preferences for ”Western”-culture products. The latter two indices could also indicate the level of infrastructure development and liberalism of policies and thus might also be correlated with the penetration of supermarkets and foreign food companies, which in turn could potentially lead to increased access to and reduced prices of NOVA 4 *vis-à-vis* NOVA 1 to 3 foods.

$Z_{\mathrm{kt}}$ comprises FAO’s international FPI and the share of the global value of NOVA 4 imports in total global NOVA 1 to 4 imports. The FPI is an index composed of international prices for 23 food commodities and has been deflated using a manufactures unit value index (FAO, 2013). Correspondingly, the FPI is a proxy measure for the global development of basic NOVA 1 food prices relative to prices of manufactured products such as NOVA 4 foods. Although NOVA 1 to 3 products are also inputs to the production of NOVA 4 foods, they likely represent only a small share of NOVA 4 production costs compared with manufacturing costs. Thus, an increase in the FPI tentatively indicates that global NOVA 1 prices rise relative to those of NOVA 4 and thus affect the price gap. Finally, the share of the global value of NOVA 4 imports in total global NOVA 1 to 4 imports is taken as an indication of the general state of competitiveness of NOVA 4 products compared with NOVA 1 to 3 products. This includes, among other factors, the technological progress in the design, production and distribution of NOVA 4 products over time, which alter consumer preferences.

Other variables have been excluded because their coefficients turned out not to be statistically significant, at least at the 10 percent level – e.g. measures of foreign direct investment – or because their data availability was insufficient – e.g. measures related to education level or physical activity.

Note that all independent variables have been lagged by one year in order to allow time for the drivers to operate and their effects to appear in the respective prevalence rate.

Potential endogeneity issues of our main variable $T_{\mathrm{it}}$ due to reverse causality between $Y_{\mathrm{it}}$ and $T_{\mathrm{it}}$ can be plausibly ruled out in reality for the following reasons: Tariff policy responses to a BMI change would necessarily be lagged substantially due to the delay with which a change in a tariff at the border might result in a measurable body weight effect, to measurement constraints typical for agriculture and health, and to the duration of the approval process to change a tariff. Moreover, as a policy instrument, tariffs are much less well-targeted at tackling obesity or undernutrition issues than consumption taxes because they often serve several other purposes, such as protecting domestic producers. Finally, most countries have only limited control over their tariffs as they are regularly tied into a multitude of trade agreements, for example, as members of the WTO, customs unions or free trade areas.

The Lagrange multiplier test for the presence of fixed effects by Honda (Baltagi, 2005), confirmed the presence of country-fixed effects (null hypothesis rejected) but absence of time-fixed effects (null hypothesis not rejected) from the baseline regression. Contrasting random versus fixed effects assumptions, using the Hausman test (Wooldridge, 2016), rejects the null hypothesis that the random effects model is consistent for the obesity model (Hausman test *p*-value = 0.019) but not for the underweight model (*p*-value = 0.578). Nevertheless, as it seems less plausible that the countries included can be considered as a random sample from a large population, we continue for both cases with country-fixed effects models estimated using ordinary least squares regression.^9^ All standard errors reported are heteroskedasticity- and serial correlation-consistent, calculated according to Arellano; see, for instance, Baltagi (2005). For completeness, random effects results for the main specifications are presented in the Appendix in Table A.2 and show no substantial differences in the results.

## 3 Results and discussion

Table 3 presents the regression results for the main analysis of the impacts of the NOVA 4 to NOVA 1 to 3 tariff difference (henceforth called dTariff) on obesity and underweight, respectively.^10^ The base specification with percentage of obesity as the independent variable is presented in column (1) alongside two robustness checks in columns (2) and (3). The base specification for the percentage of underweight is shown in column (4). Starting with column (1), most estimated coefficients are statistically different from zero at the 5 percent significance level and have the expected sign. In most cases, they are also in line with the findings from the sparse literature which, similarly to this study, utilizes regression analysis of cross-country data for estimating the effects of determinants of obesity prevalence (Goryakin et al., 2017, Loureiro et al., 2005) but with different foci and different datasets and countries. Moreover, here we also include such literature focusing on BMI (Goryakin et al., 2017, Lawson et al., 2016, Ljungvall, 2013, de Vogli et al., 2014, de Vogli et al., 2014). Although an increase in population average BMI is neither a necessary nor a sufficient condition for an increase in obesity prevalence, in most cases the two indices are likely to be moving together.

**Table 3:** Fixed effects regression results.

Dependent variable	% obesity			% under-weight
	(1)	(2)	(3)	(4)
${\text{dTariff}}_{N4-N123}$	−0.1791^∗∗∗^	−0.1805^∗∗∗^		0.0548^∗∗^
	(0.0406)	(0.0411)		(0.0179)
${\text{dTariff}}_{N34-N12}$			−0.1759^∗∗^	
			(0.0930)	
${\text{Tariff}}_{N4}$		0.0013		
		(0.0070)		
ln(GDP/capita)	2.1173^∗∗^	2.1279^∗∗^	2.2028^∗∗^	−3.2277^∗∗∗^
	(1.0716)	(1.0729)	(1.0740)	(0.6830)
ln(GDP/capita)^2^	−0.4391^∗∗^	−0.4411^∗∗^	−0.4618^∗∗^	0.4709^∗∗∗^
	(0.2156)	(0.2165)	(0.2195)	(0.1129)
FPI	0.0152^∗∗∗^	0.0152^∗∗∗^	0.0154^∗∗∗^	−0.0026^∗∗∗^
	(0.0016)	(0.0016)	(0.0016)	(0.0005)
% urban	0.1958^∗∗∗^	0.1959^∗∗∗^	0.1928^∗∗∗^	−0.1093^∗∗∗^
	(0.0409)	(0.0408)	(0.0413)	(0.0207)
% female labour participation	0.0869^∗∗∗^	0.0869^∗∗∗^	0.0892^∗∗∗^	−0.0090^·^
	(0.0270)	(0.0271)	(0.0264)	(0.0103)
% trade/GDP	−0.0124^∗∗∗^	−0.0123^∗∗∗^	−0.0124^∗∗∗^	0.0036^∗∗∗^
	(0.0033)	(0.0033)	(0.0033)	(0.0011)
% age ⩾65	0.5090^∗∗∗^	0.5101^∗∗∗^	0.5397^∗∗∗^	0.0671^∗∗^
	(0.1557)	(0.1559)	(0.1490)	(0.0388)
Information flows	0.0416^∗∗∗^	0.0415^∗∗∗^	0.0403^∗∗∗^	−0.0167^∗∗∗^
	(0.0184)	(0.0184)	(0.0186)	(0.0052)
$\text{Global}\%{\text{imports}}_{N4}$	0.2594^∗∗∗^	0.2590^∗∗∗^	0.2601^∗∗∗^	−0.0507^∗∗∗^
	(0.0323)	(0.0320)	(0.0325)	(0.0108)
${\text{dTariff}}_{N4-N123}$×LI_SSA	0.1647^∗∗^	0.1658^∗∗^	0.1623^∗∗^	−0.0526^∗∗^
	(0.0410)	(0.0412)	(0.0931)	(0.0178)
${\text{dTariff}}_{N4-N123}$×LMI_SSA	0.1738^∗∗^	0.1752^∗∗^	0.1692^∗∗^	−0.0475^∗∗^
	(0.0409)	(0.0414)	(0.0931)	(0.0181)
${\text{dTariff}}_{N4-N123}$×LI	0.0944	0.0943	0.0836	-0.1633^∗∗∗^
	(0.1216)	(0.1214)	(0.1510)	(0.0428)
${\text{dTariff}}_{N4-N123}$×LMI	0.1703^∗∗∗^	0.1705^∗∗∗^	0.1612^∗∗^	−0.0562^∗∗∗^
	(0.0405)	(0.0406)	(0.0930)	(0.0178)
${\text{dTariff}}_{N4-N123}$×UMI	0.1635^∗∗^	0.1653^∗∗^	0.1613^∗∗^	−0.0637^∗∗∗^
	(0.0456)	(0.0463)	(0.0962)	(0.0191)
${\text{dTariff}}_{N4-N123}$×HI	0.1946^∗∗∗^	0.1957^∗∗∗^	0.1765^∗∗^	−0.0605^∗∗∗^
	(0.0443)	(0.0443)	(0.0939)	(0.0184)
				
Observations	606	606	606	606
R^2^	0.7247	0.7247	0.7210	0.7033
Adjusted R^2^	0.6594	0.6587	0.6548	0.6329

Note, interaction effects for ${\text{dTariff}}_{N34-N12}$ are shown in the corresponding rows for ${\text{dTariff}}_{N4-N123}$. Source: Own computation. Symbols ${}^{\cdot }$, ${}^{*}$, ${}^{**}$, and ${}^{*\;*\;*}$ indicate coefficients significantly different from zero at level 0.1, 0.05, 0.01, and 0.001, respectively.

The effect of GDP per capita on obesity is positive at low levels but turns negative at higher levels. The coefficients of percentage of urban population, percentage of female labour participation, percentage of population age 65 and above, and the index for social globalization through information flows are all associated with increased obesity rates. Surprisingly, a higher share of trade in GDP (% trade/GDP) has a negative effect on obesity rates which also persists if this variable is replaced by an economic openness index from the KOF indices (“Actual flows”, see KOF, 2017). Thus, we find openness to international trade to be obesity-reducing. In contrast, Ljungvall (2013) and de Vogli et al. (2014) find no statistically significant effect of openness and of some “freedom to trade internationally” index, respectively. de Vogli et al. (2014) find a positive effect of economic globalization on BMI. One potential explanation is that while integration with international markets increases availability and decreases general price levels of NOVA 4 products, it does the same for less processed food options – e.g. affecting the availability and prices of vegetables out of season.

The two indicators for global developments both have the expected signs. A higher FPI, implying agricultural commodities have become more expensive relative to manufactures, is associated with higher obesity prevalence. This might be attributed to the shift in relative prices which drives consumers to substitute some of their NOVA 1 to 3 products with NOVA 4 products. Likewise, the percentage of NOVA 4 in total global NOVA 1 to 4 import value is associated with increased obesity rates.

**Fig. 1:**
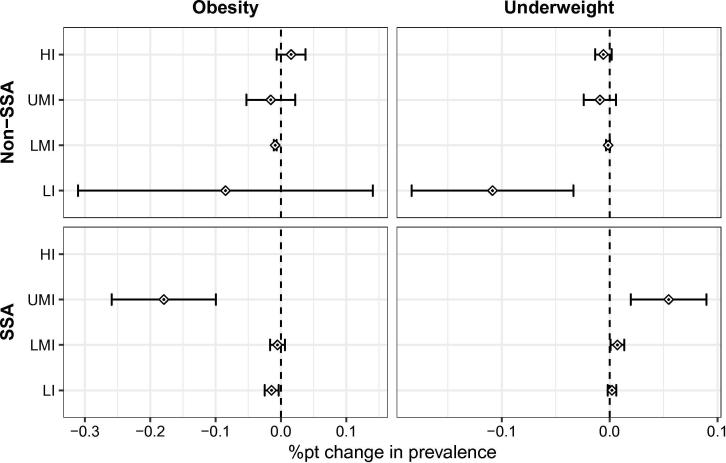
Marginal effects and their 95% confidence intervals in terms of the %pt change in obesity or underweight prevalence caused by a 1%pt increase in ${\text{dTariff}}_{N4-N123}$. Source: Own depiction.

Our main variable of interest is the dTariff variable. To allow the effect of dTariff to vary according to the state of development of the country, this variable is interacted with binary variables specifying the country-income group as defined by the World Bank and whether it is a SSA country or not, as summarized in Table 1. The reference category is upper-middle-income (UMI) in SSA which is associated with a negative effect on obesity prevalence. With the exception of the low-income (LI) non-SSA group, the interaction terms for all other country groups are statistically different from zero, indicating that the effect of dTariff in each of these country groups differs from that of UMI SSA. Such differences in the effects of BMI- and obesity-influencing factors between low- and high-income countries have been also suggested by McLaren, 2007, Swinburn et al., 2011 and Traill, Mazzocchi, Shankar, and Hallam (2014), who report that obesity prevalence is higher among the richer population in developing countries while it is higher among the poorer population in developed countries. The combined effects of dTariff and its interaction terms are presented as partial effects together with the corresponding 95 percent confidence intervals in the left panels of Fig. 1.

For LI, UMI and high-income (HI) non-SSA countries and lower-middle-income (LMI) SSA countries the estimated effects of dTariff on obesity prevalence are small and statistically not significant. In the case of UMI SSA, there is a significant and substantial negative effect of about −0.18 percentage points. This means that an increase of the difference between NOVA 4 and NOVA 1 to 3 import tariffs by one percentage point decreases the obesity prevalence rate by 0.18 percentage points. According to these estimates, for South Africa, a UMI country with a population of 53.3 million and an obesity prevalence rate of 26 percent in 2013, a 10 percentage point increase in dTariff would reduce the number of obese people by about 955 000 or 6.9 percent. The estimated effect for LI SSA countries as a group is only −0.014. However, for Uganda, a country of this group with a population of 37.6 million and an obesity prevalence rate of 4.1 percent in 2013, the same increase in dTariff would translate into a reduction of obesity prevalence by about 54 000 people or 3.5 percent.

Columns (2) and (3) of Table 3 are presented as robustness checks of the dTariff coefficient. To assess the choice of using the *difference* between NOVA 4 and NOVA 1 to 3 tariffs instead of using the tariff *levels* directly, column (2) adds a variable for the tariff level of NOVA 1 to 3. However, the variable turns out to be not statistically significant and the coefficient of dTariff changes only minimally. This provides evidence that the difference between the tariff levels as opposed to their absolute levels is indeed the decisive element to capture the effects on obesity and underweight prevalence. In column (3), the dTariff variable is replaced by a similar tariff difference variable but between NOVA 3 and 4 and NOVA 1 and 2. If both our re-classification from HS and the classification into NOVA 4 effectively delineate a group of obesity-promoting processed foods, including NOVA 3 together with NOVA 4 in the highly processed foods group for calculating the tariff difference should not change the coefficient much. This is confirmed by the coefficient of the ${\text{dTariff}}_{N34-N12}$ variable in column (3), which is only marginally larger than the ${\text{dTariff}}_{N4-N123}$ coefficient in column (1). The same holds true for the interaction terms. In sum, these results provide additional confidence in the robustness of the dTariff variable we have chosen.

Finally, the estimates in column (4) are obtained using the base specification but with underweight replacing obesity prevalence as the dependent variable. As expected, for most of the variables, the coefficients change signs with this specification (apart from ‘% age ⩾65’). All coefficients are significant at least at the 1 percent level with the notable exception of ‘% female labour participation’. Considering income level as the most important determinant of underweight, the estimated effects are as follows. At low levels of GDP per capita, an increase in income would reduce underweight the most. Afterwards, the effect diminishes continuously with increasing levels of GDP per capita. A higher percentage of urban population and improved information flows are both associated with underweight reduction. This may be explained by the fact that urbanites generally experience easier access to foods. Moreover, improved information flows imply better infrastructure and thus improved access to food in addition to better information about food availability. Contrary to expectations, a higher share of trade in GDP is linked to higher underweight prevalence rates. It could be speculated that, the poorest tend to benefit less from the gains from trade and are negatively affected by income redistribution effects compared to other income groups in a given country. However, the literature does not yet provide simple and unambiguous answers on this question (Winters & Martuscelli, 2014). Moreover, a higher percentage of population aged 65 and above is also found to be associated with higher underweight prevalence rates. It could be anticipated that the higher the share of elderly people the lower the share of the active population and therefore the lower the potential to grow food or generate income to purchase food among the poorer.

The coefficients for both FPI and global percentage of ${\text{imports}}_{N4}$ indicate an underweight-reducing effect. This could thus point to an important role of NOVA 4 products for the nourishment of the poorest, as a higher FPI implies that prices for processed foods decrease relative to those for unprocessed foods.

The calculated partial effects of the main variable ${\text{dTariff}}_{N4-N123}$ on underweight are shown in the right panels of Fig. 1. The effects in LI non-SSA countries, are found to be negative and significant. By contrast, the effects for LMI and UMI SSA countries are positive and significant. The estimated effects for the latter two groups are 0.007 and 0.055, respectively. Taking South Africa (population 53.3 million, of whom 5.1 percent are underweight) and Zambia (population 15.2 million, of whom 11.9 percent are underweight) as examples, the estimates predict that a 10 percentage point increase in dTariff would translate into increases in underweight prevalence by about 292 000 people or 10.7 percent and about 11 000 people or 0.6 percent, respectively.

Because, in the past, obesity and underweight prevalence showed markedly different developments for women and for men in SSA, we examine gender-specific effects of dTariff. For this purpose, the base specification is estimated with gender-specific obesity and underweight prevalence rates, respectively, as independent variables. The results are presented in Table 4.

**Table 4:** Results of gender-differentiated fixed effects regressions.

Dependent variable	% obesity		% underweight	
	Women	Men	Women	Men
	(1)	(2)	(3)	(4)
${\text{dTariff}}_{N4-N123}$	−0.2477^∗∗∗^	−0.1076^·^	0.0356^∗^	0.0745^∗∗∗^
	(0.0498)	(0.0487)	(0.0193)	(0.0240)
ln(GDP/capita)	3.8442^∗∗∗^	0.4647	−3.5155^∗∗∗^	−2.9689^∗∗∗^
	(1.1680)	(1.1812)	(0.7630)	(0.6399)
ln(GDP/capita)^2^	−0.6026^∗∗∗^	−0.2810^·^	0.5325^∗∗∗^	0.4137^∗∗∗^
	(0.2139)	(0.2396)	(0.1262)	(0.1070)
FPI	0.0140^∗∗∗^	0.0163^∗∗∗^	−0.0021^∗∗∗^	−0.0030^∗∗∗^
	(0.0016)	(0.0020)	(0.0005)	(0.0006)
% urban	0.3165^∗∗∗^	0.0743^∗^	−0.0795^∗∗∗^	−0.1401^∗∗∗^
	(0.0455)	(0.0455)	(0.0198)	(0.0228)
% female labour participation	0.0959^∗∗∗^	0.0732^∗∗∗^	−0.0099^∗^	−0.0079
	(0.0271)	(0.0301)	(0.0096)	(0.0124)
% trade/GDP	−0.0129^∗∗∗^	−0.0118^∗∗∗^	0.0035^∗∗∗^	0.0038^∗∗∗^
	(0.0034)	(0.0037)	(0.0010)	(0.0012)
% age ⩾65	0.2424^∗∗∗^	0.7953^∗∗∗^	0.0468^∗^	0.0879 ^∗∗∗^
	(0.1474)	(0.1794)	(0.0404)	(0.0426)
Information flows	0.0403^∗∗∗^	0.0409^∗∗∗^	−0.0137^∗∗∗^	−0.0191^∗∗∗^
	(0.0136)	(0.0259)	(0.0050)	(0.0061)
$\text{Global}\%{\text{imports}}_{N4}$	0.2412^∗∗∗^	0.2710^∗∗∗^	−0.0428^∗∗∗^	−0.0587^∗∗∗^
	(0.0316)	(0.0371)	(0.0112)	(0.0116)
${\text{dTariff}}_{N4-N123}$×LI_SSA	0.2296^∗∗∗^	0.0970^·^	−0.0333^∗^	−0.0725^∗∗∗^
	(0.0498)	(0.0496)	(0.0192)	(0.0240)
${\text{dTariff}}_{N4-N123}$×LMI_SSA	0.2368^∗∗∗^	0.1076^·^	−0.0277	−0.0678^∗∗∗^
	(0.0517)	(0.0488)	(0.0196)	(0.0242)
${\text{dTariff}}_{N4-N123}$×LI	0.1702^∗^	0.0129	−0.1717^∗∗∗^	−0.1557^∗∗∗^
	(0.1272)	(0.1255)	(0.0562)	(0.0350)
${\text{dTariff}}_{N4-N123}$×LMI	0.2382^∗∗∗^	0.0992^·^	−0.0374^∗^	−0.0755^∗∗∗^
	(0.0498)	(0.0486)	(0.0193)	(0.0239)
${\text{dTariff}}_{N4-N123}$×UMI	0.2326^∗∗∗^	0.0907	−0.0408^∗^	−0.0872^∗∗∗^
	(0.0542)	(0.0532)	(0.0199)	(0.0257)
${\text{dTariff}}_{N4-N123}$×HI	0.2602^∗∗∗^	0.1256^∗^	−0.0409^∗^	−0.0805^∗∗∗^
	(0.0522)	(0.0532)	(0.0196)	(0.0247)
				
Observations	606	606	606	606
R^2^	0.7337	0.6817	0.6810	0.6960
Adjusted R^2^	0.6705	0.6062	0.6053	0.6239

Source: Own computation. Symbols ${}^{\cdot }$, ${}^{*}$, ${}^{**}$, and ${}^{*\;*\;*}$ indicate coefficients significantly different from zero at level 0.1, 0.05, 0.01, and 0.001, respectively.

While the directions of the effects of all variables are the same as in the overall population regressions, their sizes and significance levels differ from the previous analysis as well as between women and men. On obesity prevalence, the most noteworthy differences between women and men are that, for men, the associated effect of income (GDP per capita) is not significant, and the effect of living in a city (percentage urban population) is substantially lower than for women. This suggests that the main mechanisms that are associated with obesity might be different between women and men. The results for underweight prevalence do not suggest gender differences. The sizes as well as the significance levels of the effects are largely similar between women and men.

**Fig. 2:**
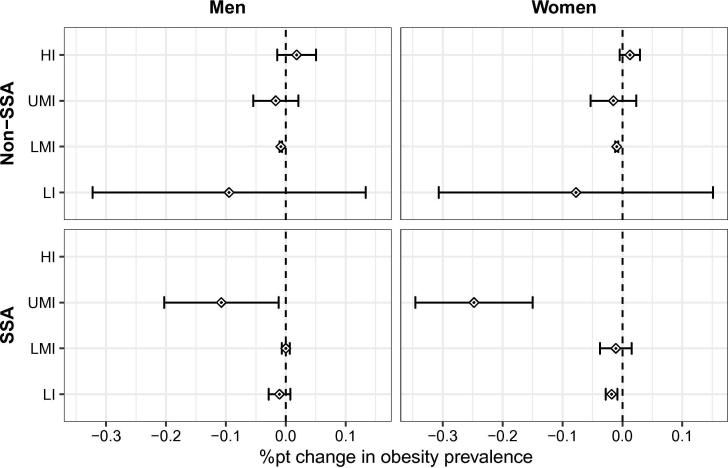
Marginal effects and their 95% confidence intervals in terms of the %pt change in obesity prevalence caused by a 1%pt increase in ${\text{dTariff}}_{N4-N123}$. Source: Own depiction.

**Fig. 3:**
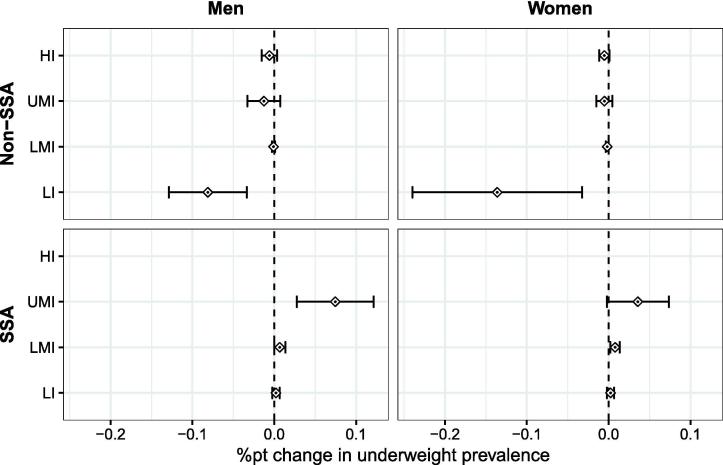
Marginal effects and their 95% confidence intervals in terms of the %pt change in underweight prevalence caused by a 1%pt increase in ${\text{dTariff}}_{N4-N123}$. Source: Own depiction.

The partial effects of dTariff on percentage of obesity and percentage of underweight from these regressions are presented in Fig. 2, Fig. 3, respectively.

In SSA, the effects of dTarriff on percentages of obesity for women are significant in LI countries (−0.02%pts) and UMI countries (−0.25%pts), at the 5 percent level. They are significant for men only in UMI countries (−0.11%pts). According to these estimates, a 10 percentage point increase in dTariff in South Africa (where 50.9% of the population is female) would reduce female obesity prevalence by about 649 000 women or 6.6 percent and reduce male obesity prevalence by about 282 000 men or 7.7 percent. An equivalent increase in dTariff in Uganda (population 37.6 million, 4.1% obese, 50% female) is predicted to reduce female obesity prevalence by about 34 000 women or 2.6 percent.

The partial effects on underweight prevalence are shown in Fig. 3. These effects in SSA are significant for women in UMI (0.04%pts) and LMI countries (0.008%pts) and for men in UMI countries (0.07%pts) at the 5 percent level. Accordingly, a 10 percentage point increase in dTariff in South Africa is expected to increase female underweight prevalence by about 93 000 women or 10.7 percent and male underweight prevalence by about 195 000 men or 10.7 percent. In the case of Zambia, classified as LMI, this amounts to an increase in female underweight by about 6 000 women or 0.83 percent.

Some limitations of this study need to be mentioned. Since sales tax and consumer price data are not comprehensively available at a global level and are even scarcer for developing countries, we have utilized import tariff data. While this approach allows us to draw direct conclusions about the effects of trade policy changes on obesity and underweight, drawing conclusions with respect to the effects of food taxes more generally requires that border and retail prices develop in constant proportions, as set out in Section 2. However, market imperfections as well as trade and transport costs exist in developing and developed countries’ markets. To the degree that these create costs which are not proportional to the product’s value at the border, the pass-through of tariffs to retail prices could be affected. Moreover, there is lack of evidence to determine how such market imperfection would change the relative price between highly and less-processed foods. Thus, to the best of our knowledge, while estimations could be less precise and standard errors for the estimates larger, we do not expect this price transmission issue to systematically bias the results into a particular direction. The prices of the imported and domestic variants are expected to be very close in the retail market as long as (i) an imported product exists which is perceived as being a close substitute for the domestic product and (ii) the price of domestic product and that of the imported one develop in similar proportion. These two conditions are considered to be reasonable assumptions.

Moreover, during the re-classification process not all HS 6-digit import tariff codes could be uniquely mapped to the four NOVA categories and thus were subject to judgement. It is recognized that this can create some degree of noise in the final panel dataset used. In addition, as noted by Gibney et al. (2017), the NOVA classification’s definition of ultra-processed foods is “too broad and too rigid” (p. 719) to define foods’ nutritional and particularly obesogenic properties. Consequently, the NOVA classification itself is a potential source of noise in the data with respect to obesity and underweight drivers. Finally, from the full dataset, we extracted a balanced panel consisting of 7 years and 101 countries including 23 countries in SSA with most of the variables desired. It is recognized that a larger dataset would yield stronger results.

Nevertheless, we are convinced that our study provides a useful contribution to the thin literature examining tax policies on processed foods to support the reduction of obesity and underweight prevalence on a global level. The novel concordance developed between the HS codes and the NOVA classification enabled us to comprehensively investigate the effects of import tariffs on all highly processed foods with respect to obesity and underweight on a global scale and also to draw some more general conclusions on what effects sales taxes might have to that end.

## 4 Conclusion

The present study contributes to the literature assessing the potential of food taxes in limiting the prevalence of obesity especially in SSA. This research adopts a new approach by mapping the import tariff data classification of the Harmonized System to the NOVA classification of food according to processing levels. It applies econometric techniques to estimate the effects on obesity and underweight prevalence in the adult population of higher import tariffs levied on highly processed compared to less processed foods.

The study finds that such tariff difference tends to be associated negatively with obesity and positively with underweight prevalence rates. However, the estimated effects are statistically significant only for particular country groups, genders and/or regions and also differ in magnitude. An additional estimation has confirmed that the absolute level of the tariff is irrelevant for these effects. Consistent with previous studies by McLaren, 2007, Nettle et al., 2017 and Swinburn et al. (2011) revealing differences in obesity developments between women and men, our results also suggest that the obesity-causing mechanisms differ by gender. The tariff difference tends to be associated with larger obesity-decreasing effects for women than for men and larger underweight-increasing effects for men than for women.

In summary, the results indicate that an increase in the tariff difference between highly processed and less processed foods can be an effective measure to reduce obesity prevalence. However, the study also suggests that such a policy needs to be applied carefully. On the one hand, while lower-income consumers might be more predisposed towards obesity this income group is also more sensitive to a rise in food prices.^11^ Thus, increasing sales taxes on highly processed foods is likely to be most effective for low income groups. On the other hand, highly processed, energy-dense foods may have become important sources of calories for the poor, in particular in urban areas. Policies aimed at making these products more expensive could push the poorest consumers further towards underweight. Moreover, considering typical properties of highly processed foods such as ease of storage, long shelf-life and high energy density, these foods might be also very important for food security in remote areas or times of scarcity.

There are several implications for policy that emerge from these results. First, decision makers willing to address overweight, obesity and underweight simultaneously are facing a policy dilemma. Hence, obesity and undernutrition cannot be treated as separate problems, particularly in developing countries. A food tax instrument as studied here is found to mitigate one problem and exacerbate another. Thus, an integrated approach, using multiple policy instruments and accounting for the side effects, is required. For instance, revenue from taxes on highly processed foods could be earmarked to fund programmes to reduce undernutrition, as discussed in Williams (2016). Such programmes would need to be well targeted at the undernourished population to avoid incentivizing additional consumption of energy-dense foods by already well-nourished individuals or reducing input costs for the production – and thus the prices – of highly processed foods themselves.

Second, any agenda to further liberalize trade, particularly in developing countries including SSA where current import tariff levels are still comparatively high, might counter the efforts to combat obesity but help reduce underweight. This is because in most countries tariffs on highly processed foods are higher than on less processed foods and any reduction of the difference in tariffs between these two groups of foods will tend to decrease underweight but, at the same time, increase obesity.

Third, the study has shown that the magnitude of the effects of the tariff differences is such that a very substantial consumption tax (or subsidy rate) would be required to meaningfully influence obesity and underweight prevalence. In our setup, the tariff difference is a percentage point difference and is equivalent to a sales tax on top of any current sales tax on highly processed foods. Our calculations for examples of SSA countries showed that a corresponding raise of a sales tax by 10 percentage points would decrease obesity prevalence by four and seven percent and increase underweight prevalence by one and 11 percent, respectively. This size of tax falls into the range of 10 to 30 percent, which is the change in retail prices that has been discussed in the literature to be effective for changing consumption of certain food groups towards healthier diets (Thow et al., 2018, WHO, 2016).

WHO (2016) also cautions that taxation of a food group or nutrient might induce consumers to substitute with other foods or nutrients which also may be unhealthy. Defining a very broad group, such as the group of highly processed foods examined here, decreases such substitution possibilities and thus will be more effective than taxes on narrower groups but will likely face strong opposition from the food industry. Moreover, the literature stresses that food taxes in general are regressive as they pose a disproportionate burden on poorer consumers (Thow, Downs, & Jan, 2014). Even though large taxes on broad food groups will be most effective, they will also put a particularly heavy burden on the poor.

In the future, additional research should be directed at the identification of food groups that can be targeted by taxes to combat obesity while avoiding detrimental effects on undernutrition. For this purpose, cross-classifying the NOVA classification with criteria related to nutrients or food uses appears to be a promising approach.

## Figures and Tables

**Table A.1:** Summary statistics of tariff data by country group.

Income group	Region	${\text{Tariff}}_{N123}$		${\text{Tariff}}_{N4}$		${\text{dTariff}}_{N4-N123}$	
		Mean	SD	Mean	SD	Mean	SD
LI	Non-SSA	7.16	3.08	14.55	6.08	7.39	3.41
	SSA	13.62	8.12	13.01	3.72	−0.61	8.99
							
LMI	Non-SSA	8.81	10.64	16.28	30.05	7.47	30.48
	SSA	11.62	7.35	15.48	6.59	3.85	7.40
							
UMI	Non-SSA	9.20	7.05	10.48	5.31	1.28	6.41
	SSA	3.77	4.80	6.43	7.18	2.66	2.73
							
HI	Non-SSA	15.91	18.32	11.51	8.54	−4.40	13.56

Source: Own elaboration. Columns show statistics on the aggregate import tariffs for the NOVA 1 to 3 (${\text{Tariff}}_{N123}$) and the NOVA 4 (${\text{Tariff}}_{N4}$) groups and the difference between these groups (${\text{dTariff}}_{N4-N123}$), respectively.

**Table A.2:** Random effects regression results.

Dependent variable	% obesity			% underweight		
	All	Women	Men	All	Women	Men
	(1)	(2)	(3)	(4)	(5)	(6)
(Intercept)	−10.5977^∗∗∗^	−8.9398^∗∗^	−12.9887^∗∗∗^	21.0874^∗∗∗^	17.8582^∗∗∗^	24.2757^∗∗∗^
	(2.5471)	(3.3974)	(2.3372)	(1.6869)	(1.5988)	(1.9040)
LI_SSA	−4.8821	−4.7335	−4.1877	−3.8096^∗^	−2.9080	−4.7487^∗∗^
	(2.6639)	(3.7705)	(2.1222)	(2.0943)	(2.1381)	(2.1920)
LMI_SSA	−5.9680^·^	−7.2672^·^	−4.0403	−2.9073	−2.3133	−3.5330^·^
	(2.3139)	(3.5391)	(1.4779)	(1.6334)	(1.4611)	(1.9041)
LI	−4.2306	−4.3539	−3.1966	−1.5955	0.9917	−4.2377
	(2.5654)	(3.6775)	(1.9905)	(3.6343)	(4.1741)	(3.2173)
LMI	−1.1598	−2.9956	1.1506	−3.9500^∗^	−1.8550	−6.0797^∗∗∗^
	(2.7591)	(3.8724)	(1.9132)	(1.7355)	(1.6256)	(1.9293)
UMI	−0.0297	−4.3576	4.4828^∗^	−4.9284^∗∗^	−2.8924^·^	−7.0039^∗∗∗^
	(2.5439)	(3.7968)	(1.5597)	(1.3353)	(1.0829)	(1.6470)
HI	−2.6207	−10.4301^∗∗∗^	5.1518^∗^	−3.6576^∗^	−1.5742	−5.8104^∗∗∗^
	(2.9633)	(4.0336)	(2.3115)	(1.4272)	(1.1578)	(1.7535)
${\text{dTariff}}_{N4-N123}$	−0.1978^∗∗∗^	−0.2641^∗∗∗^	−0.1317^∗^	0.0534^∗∗^	0.0350^∗^	0.0717^∗∗∗^
	(0.0392)	(0.0513)	(0.0388)	(0.0180)	(0.0194)	(0.0247)
ln(GDP/capita)	1.8620^∗∗^	3.5988^∗∗∗^	0.1366	−3.2846^∗∗∗^	−3.5575^∗∗∗^	−3.0529^∗∗∗^
	(0.9767)	(1.0683)	(1.0676)	(0.6992)	(0.7799)	(0.6556)
ln(GDP/capita)^2^	−0.4503^∗∗^	−0.6757^∗∗∗^	−0.1689	0.4927^∗∗∗^	0.5471^∗∗∗^	0.4477^∗∗∗^
	(0.1915)	(0.1969)	(0.2078)	(0.1139)	(0.1277)	(0.1071)
FPI	0.0173^∗∗∗^	0.0165^∗∗∗^	0.0178^∗∗∗^	−0.0026^∗∗∗^	−0.0021^∗∗∗^	−0.0031^∗∗∗^
	(0.0014)	(0.0014)	(0.0017)	(0.0006)	(0.0006)	(0.0006)
% urban	0.1756^∗∗∗^	0.2629^∗∗∗^	0.1100^∗∗∗^	−0.0983^∗∗∗^	−0.0747^∗∗∗^	−0.1193^∗∗∗^
	(0.0341)	(0.0382)	(0.0347)	(0.0185)	(0.0181)	(0.0196)
% female labour participation	0.0703^∗∗∗^	0.0795^∗∗∗^	0.0553^∗∗^	−0.0091^·^	−0.0101^∗^	−0.0080
	(0.0236)	(0.0244)	(0.0256)	(0.0100)	(0.0092)	(0.0121)
% trade/GDP	−0.0128^∗∗∗^	−0.0143^∗∗∗^	−0.0108^∗∗∗^	0.0038^∗∗∗^	0.0036^∗∗∗^	0.0041^∗∗∗^
	(0.0032)	(0.0032)	(0.0036)	(0.0011)	(0.0010)	(0.0012)
% age ⩾65	0.3199^∗∗∗^	0.1007	0.4968^∗∗∗^	0.0500^∗^	0.0379^·^	0.0569^∗^
	(0.1401)	(0.1323)	(0.1560)	(0.0377)	(0.0405)	(0.0393)
Information flows	0.0409^∗∗∗^	0.0423^∗∗∗^	0.0353^∗∗^	−0.0194^∗∗∗^	−0.0154^∗∗∗^	−0.0237^∗∗∗^
	(0.0163)	(0.0130)	(0.0217)	(0.0051)	(0.0050)	(0.0061)
Global % ${\text{imports}}_{N4}$	0.2980^∗∗∗^	0.2871^∗∗∗^	0.3016^∗∗∗^	−0.0521^∗∗∗^	−0.0432^∗∗∗^	−0.0616^∗∗∗^
	(0.0296)	(0.0291)	(0.0330)	(0.0110)	(0.0115)	(0.0117)
${\text{dTariff}}_{N4-N123}$×LI_SSA	0.1822^∗∗∗^	0.2435^∗∗∗^	0.1221^∗^	−0.0507^∗∗^	−0.0325^·^	−0.0690^∗∗∗^
	(0.0396)	(0.0514)	(0.0401)	(0.0179)	(0.0193)	(0.0247)
${\text{dTariff}}_{N4-N123}$×LMI_SSA	0.1924^∗∗∗^	0.2527^∗∗∗^	0.1321^∗^	−0.0460^∗^	−0.0270	−0.0649^∗∗^
	(0.0396)	(0.0533)	(0.0392)	(0.0184)	(0.0197)	(0.0250)
${\text{dTariff}}_{N4-N123}$×LI	0.1225	0.2010^∗^	0.0401	−0.1608^∗∗∗^	−0.1695^∗∗∗^	−0.1522^∗∗∗^
	(0.1198)	(0.1334)	(0.1067)	(0.0395)	(0.0542)	(0.0316)
${\text{dTariff}}_{N4-N123}$×LMI	0.1900^∗∗∗^	0.2559^∗∗∗^	0.1241^∗^	−0.0548^∗∗^	−0.0368^∗^	−0.0729^∗∗∗^
	(0.0392)	(0.0512)	(0.0387)	(0.0179)	(0.0194)	(0.0246)
${\text{dTariff}}_{N4-N123}$×UMI	0.1848^∗∗∗^	0.2503^∗∗∗^	0.1199 ^·^	−0.0620^∗∗∗^	−0.0401^∗^	−0.0838^∗∗∗^
	(0.0455)	(0.0562)	(0.0458)	(0.0193)	(0.0200)	(0.0263)
${\text{dTariff}}_{N4-N123}$×HI	0.2251^∗∗∗^	0.2850^∗∗∗^	0.1691^∗∗^	−0.0584^∗∗∗^	−0.0400^∗^	−0.0764^∗∗∗^
	(0.0430)	(0.0534)	(0.0450)	(0.0186)	(0.0197)	(0.0255)
						
Observations	606	606	606	606	606	606
R^2^	0.6859	0.6837	0.6627	0.6912	0.6647	0.6912
Adjusted R^2^	0.6740	0.6718	0.6500	0.6795	0.6520	0.6795

Source: Own computation. Symbols ${}^{\cdot }$, ${}^{*},{}^{**}$, and ${}^{*\;*\;*}$ indicate coefficients significantly different from zero at level 0.1, 0.05, 0.01, and 0.001, respectively.

## Footnotes

2 Recent literature reviews on economic costs of obesity are provided by Cawley, 2015, Hoque et al., 2016, Specchia et al., 2015, Tremmel et al., 2017.

3 Note that the term “highly processed food” here describes food processed intensively in a number of steps but at the same time implies specific properties of the formulation involving multiple ingredients, as detailed in Section 2.

4 We have utilized the “HS combined” nomenclature as provided by the World Bank’s World Integrated Trade Solution website (WITS, https://wits.worldbank.org/) which combines the various HS revisions over the years into a single nomenclature.

5 For example, HS line 040310 describes yoghurt but a yoghurt can be plain or contain added sugars and fruit preparations and thus could be classified either as NOVA 1 or 4.

6 Available at https://hts.usitc.gov/ for the USA and at https://publications.europa.eu/en/publication-detail/-/publication/3baa75d6-c240-11e4-bbe1-01aa75ed71a1/language-en for the EU, both accessed on October 23, 2018.

7 See Dreher (2006) for detailed descriptions of these indices.

8 The World Bank list of economies June 2017, retrieved 29 October 2017 from https://datahelpdesk.worldbank.org/knowledgebase/articles/906519-world-bank-country-and-lending-groups.

9 See Wooldridge (2016, p. 444f) for the complete line of argument.

10 The 101 country fixed-effects coefficients have been omitted for the sake of brevity as they provide no general insight.

11 The negative relationship between the price elasticity of food demand and the income level is directly implied by the well-known Engel’s Law (Clements & Chen, 1996). Empirical evidence that lower income households generally have higher price elasticities of demand for various food groups than higher income households is summarised in the meta-analysis by Green et al. (2013), for example.

## References

[b0005] Alston J.M., MacEwan J.P., Okrent A.M. (2016). The economics of obesity and related policy. Annual Review of Resource Economics.

[b0010] Asfaw A. (2008). Does supermarket purchase affect the dietary practices of households? Some empirical evidence from Guatemala. Development Policy Review.

[b0015] Augustin M.A., Riley M., Stockmann R., Bennett L., Kahl A., Lockett T., Cobiac L. (2016). Role of food processing in food and nutrition security. Trends in Food Science & Technology.

[b0020] Backholer K., Sarink D., Beauchamp A., Keating C., Loh V., Ball K., Peeters A. (2016). The impact of a tax on sugar-sweetened beverages according to socio-economic position: A systematic review of the evidence. Public Health Nutrition.

[b0025] Baltagi B.H. (2005). Econometric analysis of panel data.

[b0030] Batis C., Rivera J.A., Popkin B.M., Taillie L.S. (2016). First-year evaluation of Mexico’s tax on nonessential energy-dense foods: An observational study. PLOS Medicine.

[b0035] Botelho R., Araújo W., Pineli L. (2018). Food formulation and not processing level: Conceptual divergences between public health and food science and technology sectors. Critical Reviews in Food Science and Nutrition.

[b0040] Canella D.S., Levy R.B., Martins A.P.B., Claro R.M., Moubarac, Baraldi L.G., Monteiro C.A. (2014). Ultra-processed food products and obesity in Brazilian households (2008–2009). PLOS ONE.

[b0045] Caro J.C., Ng S.W., Taillie L.S., Popkin B.M. (2017). Designing a tax to discourage unhealthy food and beverage purchases: The case of Chile. Food Policy.

[b0050] Cawley J. (2015). An economy of scales: A selective review of obesity’s economic causes, consequences, and solutions. Journal of Health Economics.

[b0055] Clements K.W., Chen D. (1996). Fundamental similarities in consumer behaviour. Applied Economics.

[b0060] Cornelsen L., Smith R.D. (2018). Viewpoint: Soda taxes – four questions economists need to address. Food Policy.

[b0065] de Vogli R., Kouvonen A., Elovainio M., Marmot M. (2014). Economic globalization, inequality and body mass index: a cross-national analysis of 127 countries. Critical Public Health.

[b0070] de Vogli R., Kouvonen A., Gimeno D. (2014). The influence of market deregulation on fast food consumption and body mass index: A cross-national time series analysis. Bulletin of the World Health Organization.

[b0075] Demmler K.M., Ecker O., Qaim M. (2018). Supermarket shopping and nutritional outcomes: A panel data analysis for urban Kenya. World Development.

[b0080] Dreher A. (2006). Does globalization affect growth? Empirical evidence from a new index. Applied Economics.

[b0085] FAO (2013). Food outlook, Biannual report on global food markets November 2013.

[b0090] FAO (2017). Food Price Index, Statistics Division, Food and Agriculture Organization of the United Nations, Rome, Italy. Retrieved on October 27, 2017 from http://www.fao.org/worldfoodsituation/foodpricesindex/en/.

[b0095] FAO (2018). Trade and nutrition technical note, Trade Policy Technical Notes 21.

[b0100] Fardet A., Rock E., Bassama J., Bohuon P., Prabhasankar P., Monteiro C., Achir N. (2015). Current food classifications in epidemiological studies do not enable solid nutritional recommendations for preventing diet-related chronic diseases: The impact of food processing. Advances in Nutrition: An International Review Journal.

[b0105] Feenstra R.C., Taylor A.M. (2011). International macroeconomics.

[b0110] Gibney M.J., Forde C.G., Mullally D., Gibney E.R. (2017). Ultra-processed foods in human health: a critical appraisal. The American Journal of Clinical Nutrition.

[b0115] Goryakin Y., Monsivais P., Suhrcke M. (2017). Soft drink prices, sales, body mass index and diabetes: Evidence from a panel of low-, middle- and high-income countries. Food Policy.

[b0120] Goryakin Y., Rocco L., Suhrcke M. (2017). The contribution of urbanization to non-communicable diseases: Evidence from 173 countries from 1980 to 2008. Economics & Human Biology.

[b0125] Green R., Cornelsen L., Dangour A.D., Turner R., Shankar B., Mazzocchi M., Smith R.D. (2013). The effect of rising food prices on food consumption: Systematic review with meta-regression. BMJ.

[b0130] Hagenaars L.L., Jeurissen P.P.T., Klazinga N.S. (2017). The taxation of unhealthy energy-dense foods (EDFs) and sugar-sweetened beverages (SSBs): An overview of patterns observed in the policy content and policy context of 13 case studies. Health Policy.

[b0135] Hawkes C. (2005). The role of foreign direct investment in the nutrition transition. Public Health Nutrition.

[b0140] Holmes M.D., Dalal S., Sewram V., Diamond M.B., Adebamowo S.N., Ajayi I.O., Fung T.T. (2018). Consumption of processed food dietary patterns in four African populations. Public Health Nutrition.

[b0145] Hoque M.E., Mannan M., Long K.Z., Mamun A.A. (2016). Economic burden of underweight and overweight among adults in the Asia-Pacific region: A systematic review. Tropical Medicine & International Health.

[b0150] Hyseni L., Atkinson M., Bromley H., Orton L., Lloyd-Williams F., McGill R., Capewell S. (2017). The effects of policy actions to improve population dietary patterns and prevent diet-related non-communicable diseases: scoping review. European Journal of Clinical Nutrition.

[b0155] IFPRI (2016). Global Nutrition Report 2016: From Promise to Impact: Ending Malnutrition By 2030.

[b0160] Jones-Smith J.C., Gordon-Larsen P., Siddiqi A., Popkin B.M. (2012). Is the burden of overweight shifting to the poor across the globe? Time trends among women in 39 low- and middle-income countries (1991–2008). International Journal of Obesity.

[b0165] Kearney J. (2010). Food consumption trends and drivers. Philosophical Transactions of the Royal Society B: Biological Sciences.

[b0170] Kirkpatrick S.I., Raffoul A., Maynard M., Lee K.M., Stapleton J. (2018). Gaps in the evidence on population interventions to reduce consumption of sugars: A review of reviews. Nutrients.

[b0175] KOF (2017). 2017 KOF Index of Globalization, Version: April 20, 2017. http://globalization.kof.ethz.ch/.

[b0180] Lawson R.A., Murphy R.H., Williamson C.R. (2016). The relationship between income, economic freedom, and BMI. Public Health.

[b0185] Ljungvall A. (2013). The freer the fatter? A panel study of the relationship between body-mass index and economic freedom, Working Paper 2013:23.

[b0190] Loureiro M.L., Nayga R.M. (2005). International dimensions of obesity and overweight related problems: An economics perspective. American Journal of Agricultural Economics.

[b0195] Matthews A. (2014). Trade rules, food security and the multilateral trade negotiations. European Review of Agricultural Economics.

[b0200] McLaren L. (2007). Socioeconomic status and obesity. Epidemiologic Reviews.

[b0205] Monteiro C.A., Cannon G., Levy R., Moubarac J.-C., Jaime P., Martins A.P., Sattamini I. (2016). NOVA. The star shines bright. World Nutrition.

[b0210] Monteiro C.A., Moubarac J.C., Cannon G., Ng S.W., Popkin B. (2013). Ultra-processed products are becoming dominant in the global food system. Obesity Reviews.

[b0215] Moodie R., Stuckler D., Monteiro C., Sheron N., Neal B., Thamarangsi T., Casswell S. (2013). Profits and pandemics: Prevention of harmful effects of tobacco, alcohol, and ultra-processed food and drink industries. The Lancet.

[b0220] Moubarac J.-C., Martins A.P.B., Claro R.M., Levy R.B., Cannon G., Monteiro C.A. (2013). Consumption of ultra-processed foods and likely impact on human health. evidence from Canada. Public Health Nutrition.

[b0225] Moubarac J.-C., Parra D.C., Cannon G., Monteiro C.A. (2014). Food classification systems based on food processing: Significance and implications for policies and actions: A systematic literature review and assessment. Current Obesity Reports.

[b0230] NCD-RisC (2016). Trends in adult body-mass index in 200 countries from 1975 to 2014: a pooled analysis of 1698 population-based measurement studies with 19.2 million participants. The Lancet.

[b0235] Nettle D., Andrews C., Bateson M. (2017). Food insecurity as a driver of obesity in humans: The insurance hypothesis. Behavioral and Brain Sciences.

[b0240] Ng M., Fleming T., Robinson M., Thomson B., Graetz N., Margono C., Biryukov S. (2014). Global, regional, and national prevalence of overweight and obesity in children and adults during 1980–2013: A systematic analysis for the Global Burden of Disease Study 2013. The Lancet.

[b0245] Niebylski M.L., Redburn K.A., Duhaney T., Campbell N.R. (2015). Healthy food subsidies and unhealthy food taxation: A systematic review of the evidence. Nutrition.

[b0250] Popkin B.M., Adair L., Ng S.W. (2012). Global nutrition transition and the pandemic of obesity in developing countries. Nutrition Reviews.

[b0255] Popkin B.M., Gordon-Larsen P. (2004). The nutrition transition: Worldwide obesity dynamics and their determinants. International Journal of Obesity.

[b0260] Reardon T., Timmer C.P., Barrett C.B., Berdegue J. (2003). The rise of supermarkets in Africa, Asia, and Latin America. American Journal of Agricultural Economics.

[b0265] Regmi A., Gehlhar M.J., Wainio J., Vollrath T.L., Johnston P.V., Kathuria N. (2005). Market access for high-value foods, Agricultural Economic Report 840.

[b0270] Specchia M.L., Veneziano M.A., Cadeddu C., Ferriero A.M., Mancuso A., Ianuale C., Ricciardi W. (2015). Economic impact of adult obesity on health systems: a systematic review. European Journal of Public Health.

[b0275] Stacey N., Tugendhaft A., Hofman K. (2017). Sugary beverage taxation in South Africa: Household expenditure, demand system elasticities, and policy implications. Preventive Medicine.

[b0280] Stuckler D., McKee M., Ebrahim S., Basu S. (2012). Manufacturing epidemics: The role of global producers in increased consumption of unhealthy commodities including processed foods, alcohol, and tobacco. PLOS Medicine.

[b0285] Swinburn B., Sacks G., Hall K.D., McPherson K., Finegood D.T., Moodie M.L., Gortmaker S.L. (2011). The global obesity pandemic: Shaped by global drivers and local environments. The Lancet.

[b0290] Thow A.M. (2009). Trade liberalisation and the nutrition transition: mapping the pathways for public health nutritionists. Public Health Nutrition.

[b0295] Thow A.M., Downs S., Jan S. (2014). A systematic review of the effectiveness of food taxes and subsidies to improve diets: Understanding the recent evidence. Nutrition Reviews.

[b0300] Thow A.M., Downs S.M., Mayes C., Trevena H., Waqanivalu T., Cawley J. (2018). Fiscal policy to improve diets and prevent noncommunicable diseases: From recommendations to action. Bulletin of the World Health Organization.

[b0305] Thow A.M., Jan S., Leeder S., Swinburn B. (2010). The effect of fiscal policy on diet, obesity and chronic disease: A systematic review. Bulletin of the World Health Organization.

[b0310] Townsend R., Jaffee S., Hoberg Y., Htenas A., Shekar M., Hyder Z., Elder L. (2016). Future of food: Shaping the global food system to deliver improved nutrition and health.

[b0315] Traill W.B. (2017). Transnational corporations, food systems and their impacts on diets in developing countries. Trade Policy Technical Notes 17.

[b0320] Traill W.B., Mazzocchi M., Shankar B., Hallam D. (2014). Importance of government policies and other influences in transforming global diets. Nutrition Reviews.

[b0325] TRAINS (2017). Trade Analysis Information System (TRAINS) Database, United Nations Conference on Trade and Development, Geneva, Switzerland. Retrieved via WITS on September 15, 2017 from http://wits.worldbank.org.

[b0330] Tremmel M., Gerdtham U.-G., Nilsson P.M., Saha S. (2017). Economic burden of obesity: A systematic literature review. International Journal of Environmental Research and Public Health.

[b0335] UN COMTRADE (2017). United Nations Commodity Trade Statistics Database (COMTRADE), Statistics Devision, United Nations Conference on Trade and Development, Geneva, Switzerland. Retrieved on October 13, 2017 from https://comtrade.un.org/data/.

[b0340] WDI (2017). World Development Indicators, The World Bank, Washington, DC. Retrieved on September 9, 2017 from http://data.worldbank.org/data-catalog/world-development-indicators.

[b0345] WHO (2003). Diet, nutrition and the prevention of chronic diseases. WHO Technical Report Series 916.

[b0350] WHO (2016). Fiscal policies for diet and prevention of noncommunicable diseases, Technical meeting report, 5–6 May 2015.

[b0355] Williams S.N. (2016). ‘Soda taxes’ and ‘fat taxes’ can help tackle the twin problems of global obesity and under-nutrition. Perspectives in Public Health.

[b0360] Winters L.A., Martuscelli A. (2014). Trade liberalization and poverty: What have we learned in a decade?. Annual Review of Resource Economics.

[b0365] Wooldridge J.M. (2016). Introductory econometrics: A modern approach.

[b0370] Wright A., Smith K.E., Hellowell M. (2017). Policy lessons from health taxes: a systematic review of empirical studies. BMC Public Health.

[b0375] Ziraba A.K., Fotso J.C., Ochako R. (2009). Overweight and obesity in urban Africa: A problem of the rich or the poor?. BMC Public Health.

